# Oral community health worker-led interventions in households with average levels of psychosocial factors

**DOI:** 10.3389/froh.2022.962849

**Published:** 2022-08-11

**Authors:** Helen H. Lee, David Avenetti, Yuwa Edomwande, Vyshiali Sundararajan, Liyong Cui, Michael Berbaum, Rachel Nordgren, Anna Sandoval, Molly A. Martin

**Affiliations:** ^1^Department of Anesthesiology, University of Illinois at Chicago, Chicago, IL, United States; ^2^Institute for Health Research and Policy, University of Illinois at Chicago, Chicago, IL, United States; ^3^Department of Pediatric Dentistry, University of Illinois at Chicago, Chicago, IL, United States; ^4^Department of Pediatrics, University of Illinois at Chicago, Chicago, IL, United States

**Keywords:** community health worker, oral health, psychosocial stress, childhood, parenting, social determinants of health

## Abstract

**Introduction:**

Household-level psychosocial stress levels have been linked to child tooth brushing behaviors. Community health worker (CHW) interventions that target psychosocial factors in high-risk communities have been associated with changes in health behaviors.

**Aim:**

Observe changes in psychosocial factors over time and an association between psychosocial factors and CHW intervention dose amongst urban Chicago families.

**Patients and methods:**

Participants (*N* = 420 families) were recruited from 10 community clinics and 10 Women, Infants, or Children (WIC) centers in Cook County, Illinois to participate in a clinical trial. Research staff collected participant-reported psychosocial factors (family functioning and caregiver reports of depression, anxiety, support, and social functioning) and characteristics of CHW-led oral health intervention visits (number, content, child engagement) at 0, 6, and 12 months. CHWs recorded field observations after home visits on household environment, social circumstances, stressors, and supports.

**Results:**

Participants across the cohort reported levels of psychosocial factors consistent with average levels for the general population for nearly all measures. Psychosocial factors did not vary over time. Social functioning was the only measure reported at low levels [32.0 (6.9); 32.1 (6.7); 32.7 (6.9); mean = 50 (standard deviation)] at 0, 6, and 12 months. We did not observe a meaningful difference in social functioning scores over time by exposure to CHW-led intervention visits (control arm, 0, 1, 2, 3, and 4 visits). Field observations made by CHWs described a range of psychosocial stress related to poverty, language barriers, and immigration status.

**Conclusion:**

The unexpectedly average and unchanging psychosocial factors over time, in the context of field observations of stress related to poverty, lack of support, immigration status, and language barriers, suggests that our study did not adequately capture the social determinants of health related to oral health behaviors or that measurement biases precluded accurate assessment. Future studies will assess psychosocial factors using a variety of instruments in an attempt to better measure psychosocial factors including social support, depression, anxiety, functioning, trauma and resilience within our urban population. We will also look at neighborhood-level factors of community distress and resilience to better apply the social ecologic model to child oral health behaviors.

## Introduction

Early childhood caries is the most common chronic disease of childhood and persists as a source of racial/ethnic inequity in disease burden. The etiology of caries development is multifactorial, but largely influenced by caregiver and child health behaviors. Community health workers (CHWs) have demonstrated tremendous promise as a workforce to lead behavioral interventions by targeting social support, self-efficacy, self-management skills, and disease knowledge [1–4]. In addition to the aforementioned behavioral targets, CHWs represent a workforce that can address psychosocial factors within households that may impact behavior change [1].

Coordinate Oral Health Promotion (CO-OP) Chicago was a cluster-randomized behavioral trial that targeted oral health knowledge and self-management skills with CHWs to change oral health behaviors in young children [5]. CO-OP focused on urban households in the Chicago area. The primary outcomes of the clinical trial were children's tooth brushing, measured using self-reported frequency and observed plaque score. The intervention, delivered by CHWs mainly in homes, was not associated with a difference in brushing when compared to brushing in a wait-list control group [6]. This result was different than expected and did not align with other CHW research. Therefore, we conducted secondary analyses to determine why the intervention did not lead to changes in oral health behaviors.

Social ecologic theory emphasizes individual, interpersonal, organizational, community, and public health factors in relation to health behavior change [7, 8]. Caregiver psychosocial stress, frequently captured as depression or anxiety, is known to impair responsiveness to behavioral interventions and has been linked to child tooth brushing behaviors [9–13]. CHW interventions support behavior changes through the pathways of social support, navigation, and advocacy [14]. Although they are not clinicians, CHWs can also assist families to address social issues contributing to psychosocial stress and navigate families to clinical mental health care [15, 16]. We tested if the trial's limited changes in oral health behaviors could be explained by the burden of psychosocial stressors borne by CO-OP caregivers. We also questioned if the CHW intervention sufficiently changed intermediary social support targets.

CO-OP's CHW intervention was based upon Bandura's Social Cognitive Theory [17]. We hypothesized that tooth brushing behaviors could be influenced by immediate feedback from CHWs that are external to the family but aligned with the participant's social network [6]. Tooth brushing behaviors, including parental supervision, have been linked to self-efficacy [18, 19]. Per Bandura, self-efficacy is developed through mastery experiences, vicarious experiences, verbal persuasion, and physiological feedback from emotional states. The CO-OP CHW intervention targeted self-efficacy using social support and education to build mastery of child oral health behaviors. CHWs also were trained to address social determinants of oral health, such as poverty, access to care, and immigration status, which have been linked to children's oral health behaviors and oral health status [20–22]. This secondary analysis was conducted to explore whether CHW-led oral health intervention was associated with psychosocial stress and social support in an urban Chicago-area population. This study tested the hypothesis that a CHW-led oral health intervention was associated with changes in caregiver and household level psychosocial factors (stressors and social support) amongst urban Chicago families. The study objectives include observing (1) changes in psychosocial factor levels over time and (2) an association between psychosocial factors and CHW intervention dose.

## Methods

### Study population

Coordinated Oral Health Promotion (CO-OP) Chicago was a cluster-randomized controlled trial that evaluated the impact of CHWs on tooth brushing for low-income urban children under 3 years old. Participants (*N* = 420 families) were recruited from 10 community clinics and 10 Women, Infants, and Children (WIC) centers in Cook County, Illinois from January 2018 to February 2019. Half (*N* = 211) were randomized to receive four oral health CHW home visits over 12-months. Study design, protocol, and measures have been previously described [5].

### Covariates

Research assistants collected participant data at 0, 6, and 12 months he Confusion, Hubbub, and Order Scale (CHAOS) is a validated measure of family functioning [23]. Caregiver-level psychosocial factors were assessed using Patient-Reported Outcomes Measurement Information System (PROMIS) measures [24]. Specific domains included depression, anxiety, social support (emotional, informational, instrumental), and social functioning (ability to participate in social roles and activities). The number of CHW visits completed constituted both an intervention dose and an assessment of the family's capacity to make and keep appointments. After completion of each visit, CHWs recorded details of the visit (covered content, participation level, number of children/adult participants, action plan) in a database. If a child was present during the visit, participation level was categorized by the degree of engagement with the CHW (A lot; A little; Not at all).

### CHW intervention

**Figure 1:**
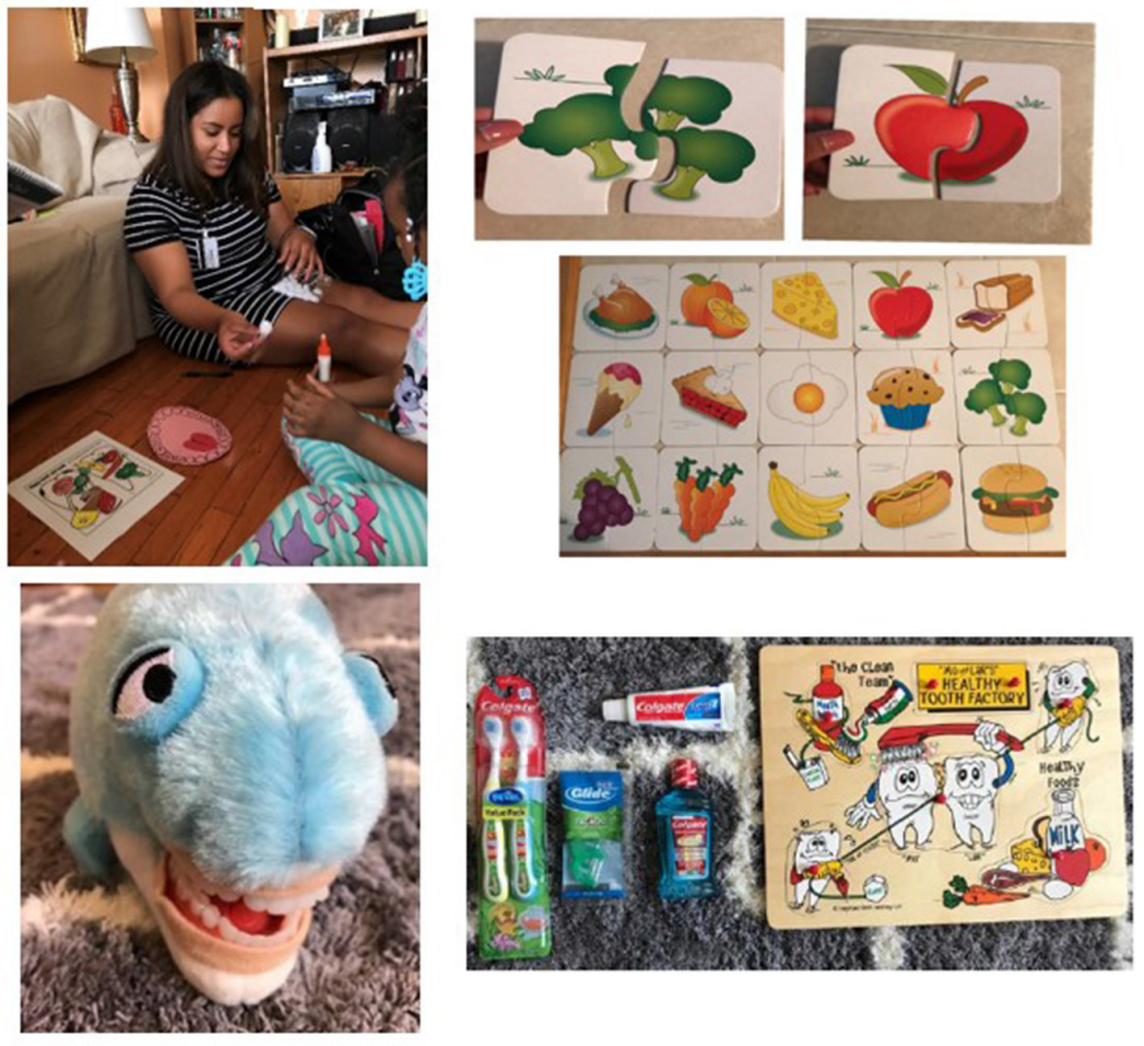
Child-based oral health education. Oral community health worker-led interventions included game or play-based activities that related to oral health topics such as tooth brushing technique and dietary choices.

CHW-led intervention included up to four visits over 1 year. There was social proximity between the CHWs and the study participants [6]. CO-OP's Spanish speaking families were paired with a Spanish-speaking CHW. The demographics of the four CHWs were as follows: female, ages 26–33 years, and two identified as Latina (Spanish-English bilingual), one as African American, and one as West African. At most first visits, CHWs conducted a Caries Risk Assessment of the participant child and caregiver [25]. Information from the Caries Risk Assessment was used to guide the CHW intervention content delivered. After each visit, CHWs reached out to caregivers through a follow-up phone call. CHWs used social cognitive theory to help families identify and make changes in oral health behaviors [17]. CO-OP CHWs applied formal self-management skills (problem solving, decision making, resource utilization, patient/doctor partnership, and taking action) to activities aimed to address the oral health core curriculum topics (basic tooth anatomy, pathological factors, early childhood caries, tooth brushing basics, fluoride basics, nutrition, oral health recommendations) [26–29]. Psychosocial health training for CHWs included mental health first aid and motivational interviewing techniques. A clinical psychologist supervised psychosocial health training. The psychologist met with CHWs every 2 months or more frequently if needed throughout the study period, to resolve mental health concerns related to CHW experiences (household/environmental stressors, poverty-related issues, occasional participant safety issues). When CHWs identified a barrier to delivery of oral health education, CHWs facilitated caregivers in incorporating a relevant self-management skill and creating an Action Plan. CHWs helped caregivers create a list of problems from which the families created their Action Plans. At subsequent CHW visits and follow-up telephone calls, CHWs reviewed past Action Plans and revised or created new ones. When a child was present during a visit, CHWs devoted time to child-based oral health education through games and activities (Figure 1). There was an option to document clinical findings (e.g., visible cavities/fillings, white spots/enamel defects), depending upon comfort levels of the CHWs and family. CHWs recorded observations in a journal after conducting home visits but entries were not tied to participant identifiers.

We measured intervention dose as the number of completed visits per family over the study period. CHWs were encouraged to maintain a journal to process their impressions from the field. These field notes were reviewed and discussed in meetings with CHWs and investigators.

### Human subjects

Institutional Review Boards at the University of Illinois at Chicago (2017-1090), the University of California San Francisco (16-19920), and the Chicago Department of Public Health (16-06) approved the trial. Caregivers provided written informed consent. Trial oversight was also provided by a Data Safety Monitoring Board, an external monitor reporting to the funder, and a Community Advisory Board.

### Analysis

PROMIS measures are reported using T-scores, where 50 is the mean for the validation reference population and SD is 10; higher scores represent more of the concept being measured. Minimal important change (MIC) is the within-person change over time in which a person's experience of the measured domain is perceived to have importantly changed. MIC values of 2–6 points are reported for non-surgical interventions [30]. Because there was no difference in oral health behaviors between participants who received and did not receive CHW intervention, psychosocial factor changes over time were plotted for the combined full cohort. To determine if psychosocial factors were associated with CHW intervention, CHW visits were first organized using descriptive methods. Data were organized to show the frequency that individual participants discussed various topics as well as the total number and frequency of topics over the study period (visits #1-4). We plotted PROMIS social functioning scores over time, stratified by control vs. intervention arms as well as the number of completed CHW visits (0, 1, and 2–4). Psychosocial factor scores are reported as means with standard deviations, as well as median with range and interquartile range (representing 1st and 3rd quartiles). We did not conduct advanced analyses on either psychosocial factor changes over time or CHW dose associations because of the limited variability observed. All data analyses were performed using SAS/STAT Version 9.4 (Cary, NC, USA).

## Results

### Study participants

CO-OP participants included 420 children and one of their caregivers. The average child age was 21.6 months (SD 6.9). Families were mainly low-income and Hispanic ethnicity or non-Hispanic Black race. We have reported demographic characteristics of CO-OP participants in a previous publication [5].

### Intervention delivery

**Figure 2:**
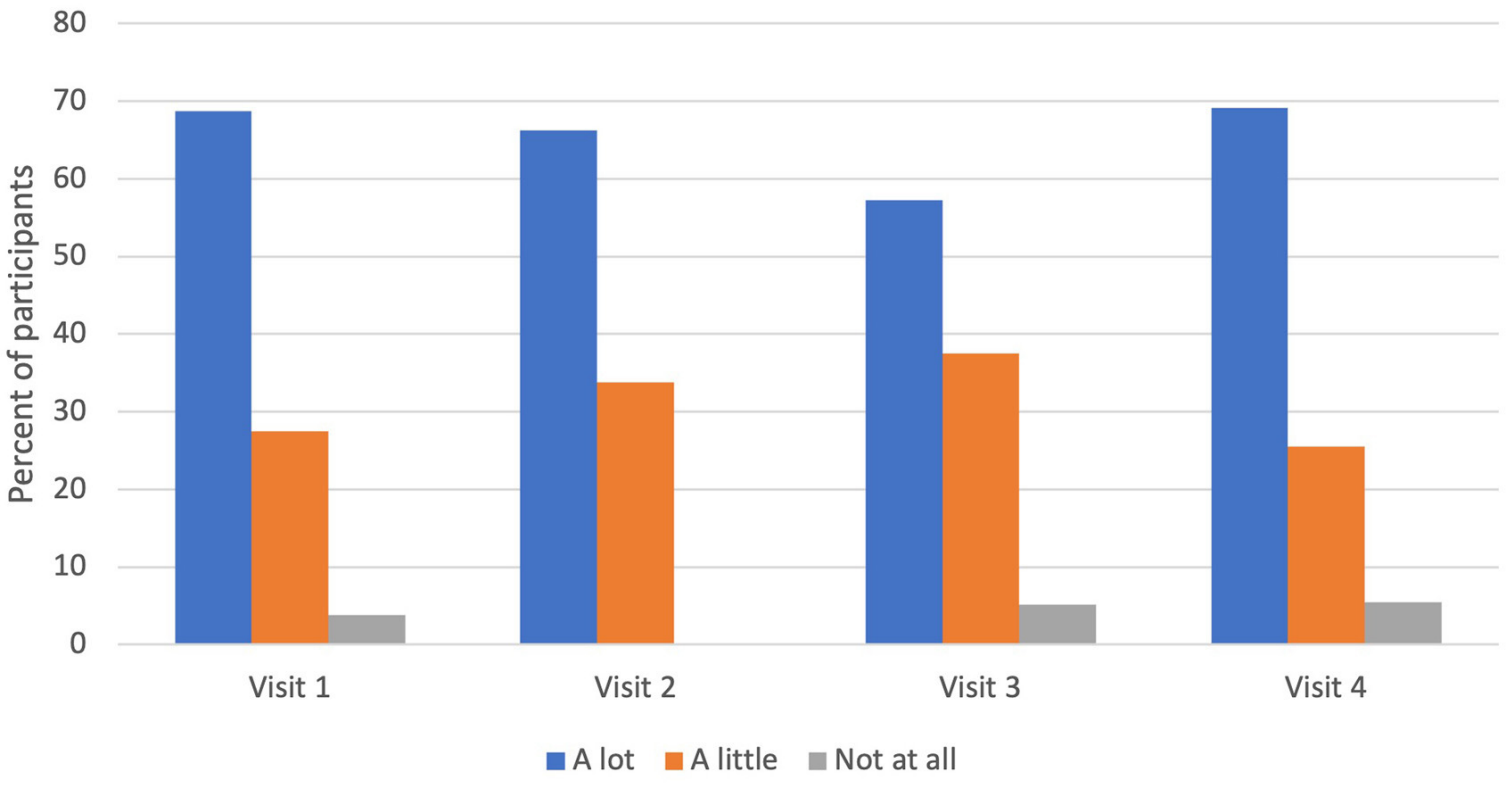
Child participation during CHW intervention visit. Child participation during a community health worker (CHW) visit remained consistent across time (visits #1-4). Majority of children, if present during a CHW visit, engaged with the CHW a lot.

Of the 420 households in the study, 211 were randomized to intervention. Interventions occurred between April 2018 and February 2020. A total of 420 CHW intervention visits were completed in this period, involving 365 different children and adults. Mothers were the predominant adult who participated in CHW visits (*N* = 387, 92.1%). Intervention visits were also attended by other participants including fathers (*N* = 56, 13.3%), sisters (*N* = 70, 16.7%), brothers (*N* = 56, 13.3%), aunt/uncle (*N* = 17, 4.0%), grandparents (*N* = 35, 8.3%), cousin (*N* = 11, 2.6%), or others who were in the household at the time of intervention (caregiver friend, great grandma, step-dad, god-sister, caregiver's partner, guardian, unknown, *N* = 18, 4.3%). Children participated in 347 intervention visits (83% of total intervention visits). Child engagement in intervention activities remained high for majority of children throughout all four visits (Figure 2).

Nearly a quarter of participants in the intervention arm received all four visits (23.7%); 12.8% received three visits; 21.3% received two visits; 23.2% received one visit; and 19.0% of participants in the intervention arm received no visits during the study period [6]. CHW visits ranged in duration from 9 to 195 min, with a mean duration of 63.7 (SD 21.8) min [6]. After a CHW visit was completed, a follow-up call was attempted. Receipt of follow-up calls were as follows: 8.5% received four, 10.9% received three, 19.0% received two, 28.4% received one, and 33.25% of intervention-arm participants received no follow up calls over a 12-month period. While visits were predominantly conducted in participant homes (*N* = 391, 93.1%), participants requested a few other alternate locations e.g., clinic (*N* = 1, 0.2%), WIC (*N* = 9, 2.1%), and Other which included grandparent home, public libraries, district park, supermarket, and tattoo shop (*N* = 19, 4.5%).

### Intervention content

Caregivers and CHWs discussed topics that were of highest interest for each household. Nearly 100% of participants discussed oral health basics, tooth brushing, and fluoride with CHWs (Table 1, see Supplement for Subtopics). Oral health behaviors such as bottle weaning (87.1%), nutrition (88.9%) and dental visits (95.3%) were also identified as important and frequently covered oral health topics. In the context of their child's oral health, caregivers and CHWs discussed other social determinants of oral health, such as insurance status, immigration, financial assistance, mental health, housing, and childcare (Table 1; Supplemental Table 7).

**Table 1:** Oral health topics covered by oral community health workers.

**Topics**	**Total participants**	**Total number of**
	**that received**	**times topic addressed**
	**topic at least**	**in visits #1-4**,
	**once, *N =* 171 (%)**	***N =* 420 (%)**
Oral health basics	169 (98.8)	261 (62.1)
Tooth brushing	169 (98.8)	382 (90.9)
Fluoride	169 (98.8)	324 (77.1)
Weaning from	149 (87.1)	259 (61.7)
bottle at night
Nutrition	152 (88.9)	318 (75.7)
Dental visit	163 (95.3)	352 (83.8)
Other topics*	132 (77.2)	261 (62.1)

^*^Other topics that participants discussed with community health workers included: insurance coverage, immigration, financial assistance, mental health, housing, childcare, child support, health/medical concerns, physical activity, and social resources.

The greatest proportion of participants, during CHW visits, discussed issues related to tooth brushing and fluoride, *N* = 169 (98.8%). Fluoridated water was the most covered oral health subtopic, reaching 169 (98.8%) participants. Other predominant subtopics included brushing frequency, frequency of foods/drinks, dental visit frequency, and spontaneously arising subtopics. CHWs were trained to allow participants to discuss social issues that they felt were related to their children's oral health, which arose for 132 (77.2%) participants and addressed a total of 261 times over the course of visits #1-4 (62.1%). Topics reflected larger social determinants of oral health, including insurance coverage, immigration, financial assistance, mental health, housing, childcare, child support, health/medical concerns, physical activity, and social resources [31].

### Psychosocial factors

**Figure 3:**
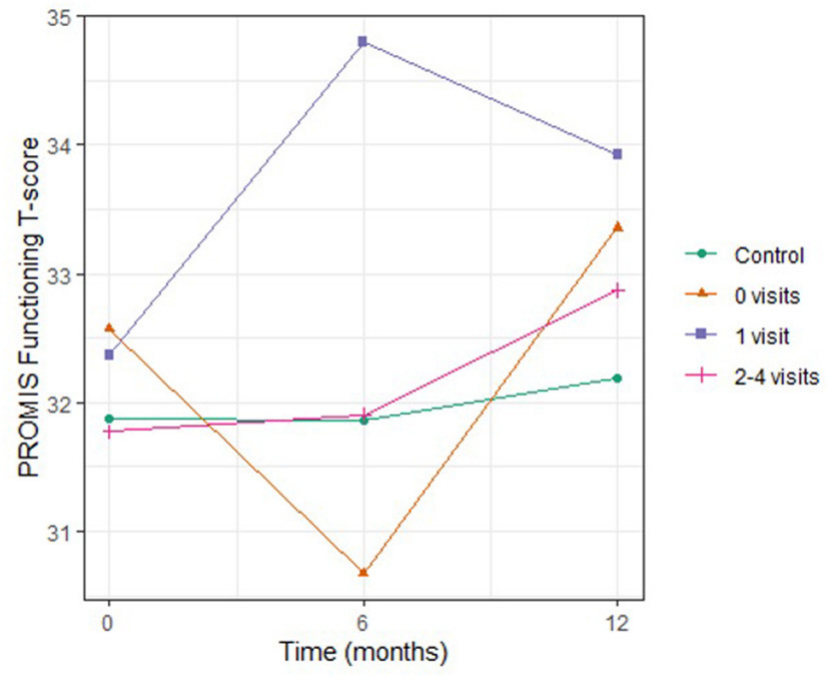
Social Functioning Levels Over Time in Urban Chicago Households with Young Children, by Number of Community Health Worker Visits. Scores for PROMIS social functioning were plotted over 12 months for study participants. Participants in the control arm did not receive any community health worker (CHW)-led interventions. Amongst participants who were in the intervention arm, PROMIS scores for social functioning did not meaningfully change over time. Stratifying by number of CHW visit received did not yield significant differences in PROMIS social functioning scores between groups nor were there differences in trends over time.

Psychosocial factor levels did not vary over time (Table 2) or differ by arm. Stress and social support levels were comparable to the general population [31], except for social functioning. CO-OP caregivers reported social functioning levels at nearly two standard deviations below the general population average [32.0 (SD 6.9), 32.1 (6.7), and 32.7 (6.9) at 0, 6, and 12 months, respectively; normal = 50 (SD 10)]. Stratifying social functioning by CHW dose (number of visits) did not reveal a dose effect; participants who had zero (33.4, SD 6.6**)** and one CHW visit (33.9, SD 6.3) reported higher social functioning than those with two (32.5, SD 7.1), 3 (33.2, SD 6.9) and three-to-four (33.0, SD 8.3) visits at 12 months (Figure 3). As there was no variation in psychosocial variables, we did not conduct further analyses.

**Table 2:** Caregiver and household psychosocial stress levels over time.

	**Baseline** ***N =* 422**	**6 months** ***N =* 366**	**12 months** ***N =* 362**
PROMIS Anxiety T-score, mean (SD); median (range, IQR)	46.6 (8.1); 40.3 (40.3–77.9, 13.4)	46.7 (8.4); 40.3 (40.3–81.6, 13.4)	46.9 (8.2); 40.3 (40.3–81.6, 13.4)
PROMIS Depression T-score, mean (SD); median (range, IQR)	46.2 (6.9); 41.0 (41.0–71.2, 10.8)	45.7 (6.8); 41.0 (41.0–79.4, 8.0)	45.7 (6.5); 41.0 (41.0–69.4, 10.8)
PROMIS Social functioning T-score, mean (SD); median (range, IQR)	32.0 (6.9); 31.3 (25.9–58.2, 10.3)	32.1 (6.7); 31.3 (25.9–55.7, 11.0)	32.7 (6.9); 31.3 (25.9–58.2, 11.8)
PROMIS Emotional T-score, mean (SD); median (range, IQR)	55.9 (8.9); 57.8 (24.7–63.5, 14.3)	56.0 (8.8); 60.7 (32.5–63.5, 14.3)	56.6 (8.3); 63.5 (24.7–63.5, 14.3)
PROMIS Informational T-score, mean (SD); median (range, IQR)	57.7 (9.8); 58.7 (27.1–69.1, 17.9)	58.0 (10.0); 58.7 (23.7–69.1, 19.0)	59.1 (9.5); 60.3 (31.8–69.1, 16.7)
PROMIS Instrumental T-score, mean (SD); median (range, IQR)^a^	54.8 (9.3); 55.4 (31.1–65.6, 18.4)	55.2 (9.4); 55.4 (31.1–65.6, 18.4)	55.5 (9.5); 55.4 (27.0–65.6, 18.4)
CHAOS Total (avg), mean (SD); median (range, IQR)	2.3 (0.6); 2.2 (1.0–4.5, 0.8)	2.3 (0.6); 2.2 (1.0–4.2, 1.0)	2.3 (0.6); 2.3 (1.0–4.3, 1.0)

*a: N = 419 participants completed PROMIS Informational survey at baseline; PROMIS T-score: 50 is the mean of a relevant reference population with 10 as SD. Higher scores represent more of the concept being measured. Minimal important change (MIC) is the within-person change over time in which patients experience of the measured domain is perceived to have importantly changed. MIC values of 2-6 points are reported for non-surgical interventions. SD, Standard Deviation; IQR, interquartile range*.

### CHW observations

CHWs recorded interactions and observations after intervention visits. While CHW observations were not hypothesis driven, we present them as a type of ethnographic data to compare with the main outcomes data. Journal entries illustrated a degree of environmental or psychosocial stress that sometimes contradicted the quantitative psychosocial data. Despite self-reported levels of anxiety, depression, or social support that was consistent with average levels for the general population, CHWs observed that caregivers battled a high degree of psychosocial stress as they tried to navigate their lives and care for their children.

CHWs were trained to facilitate navigation through the health care system (e.g., assisting with scheduling dental appointments). They encountered caregiver issues related to lack of resources and/or support to overcome barriers related to poverty; these complicated seemingly simple tasks, such as getting medicine on a rainy day.

“[On a rainy day while visiting caregiver of twin babies] *I picked up the medicine for caregiver since giving her a ride is not allowed. I gave her assistance for transportation and gave her my umbrella. Caregiver expressed the hardships of getting transportation to appointments*.”

The political climate around citizenship status and possible deportation amplified anxiety for several caregivers, which occasionally represented a barrier to health insurance.

“*Caregiver canceled kids insurance due to everything going on with immigration and what she saw on the news pertaining people having and or requesting medical card or link card for their children*.”

In the midst of conversations about oral health, caregivers shared other stressors, such as financial insecurity, language barriers, and a general lack of instrumental and informational support. While CHWs were instructed to focus on oral health topics, home-based visits also facilitated discussions related to these psychosocial stressors. Financial stress was amplified in the absence of adequate support networks and language barriers.

“…*her major worry now was the fact that bills kept piling up…Being desperate and not knowing what to do, whom to contact and speak to regarding her balance due to her bills being in a collection department… her greatest uncertainty and worry was [related to] language barrier*.”

## Discussion

Our study population reported poor levels of caregiver social functioning that did not change over time, suggesting a determinant of health not sufficiently addressed by our CHW-led intervention. Caregivers reported other psychosocial factors at levels consistent with the general population, which was unexpected. Additionally, there was no variation in the levels of psychosocial factors over time. Together, the unexpectedly normal levels and unchanging nature of psychosocial factors over time suggests that the study did not adequately capture or address the social determinants of health associated with oral health behaviors. Our psychosocial measures, while well-validated in national samples, may not capture these factors well in urban low-income populations of caregivers with young children. PROMIS instruments are vulnerable to differential item functioning, which is a measurement and item bias that could lead to individuals responding to questions as a function of race/ethnicity or other variables rather than as a function of the domain [32]. Our qualitative data suggest families did face a range of important psychosocial stressors, especially related to limited social support, and that CHWs were able to provide some assistance in these areas.

We did not observe any impact of a CHW-led behavioral intervention on caregiver and household psychosocial factors in urban Chicago families. This suggests either our intervention was not effective in moving the intermediate psychosocial targets frequently addressed by CHWs [16] or that the measurement biases (e.g., differential item functioning) precluded accurate assessment. While CHWs have been associated with changes in anxiety and depression, possibly by providing support through coaching, advocacy, and healthcare navigation, the intensity and focus of these activities relative to health education may be critical [16, 33–35]. Future studies will assess psychosocial factors using a variety of instruments in an attempt to better measure psychosocial factors including social support, depression, anxiety, functioning, trauma and resilience within our urban population. We will also look at neighborhood-level factors of community distress and resilience to better apply the social ecologic model to child oral health behaviors.

The CHW intervention content addressed education, health knowledge, and self-efficacy; the delivery was informed by social cognitive theory, which emphasized behavior change. Other CHW interventions have been designed to more heavily address psychosocial stress or prioritize social determinants of health over disease states [16, 36, 37]. Our CHWs addressed psychosocial stressors relative to oral health behaviors. Future work should address whether a CHW-led intervention would be more effective if content and dose focused more on social determinants, with a sub-emphasis on oral health. Further work should also discriminate between clinical depression and anxiety vs. experiencing psychosocial stress related to living in poverty. While psychiatric diagnoses require treatment from clinicians, mitigating social determinants of health is not a clinical intervention. It is possible that our findings of relatively normal levels of psychosocial stress reflect subclinical levels of stress. Additionally, our psychosocial measures may not reflect domains of social hardship related to living in the context of structural racism [38, 39]. An individual's concept of stress may be relative to the immediate neighborhood or community, which in this case may have led to normalization of psychosocial stress that does not represent the general population. Perhaps a pragmatic approach would be to focus on functioning, as caregivers may balance stressors of poverty and racism with coping mechanisms that result in resilience. The balance between psychosocial stress and resilience and functioning is likely to vary across time as well as across households and may represent an important factor in changing and maintaining oral health behaviors [40–42].

A possible limitation to this study is the differential receipt of intervention dose, or CHW services. While the study design included a standardized intervention (four CHW visits offered to each family in the CHW group), uptake of a behavioral intervention in the home setting also relied upon family participation. The majority of families did not choose the full 4-dose intervention. This was expected and is comparable with other CHW-led behavioral and health care interventions [43, 44]. We conducted per-protocol analyses to determine if more or less receipt of CHW visits was associated with psychosocial factors and behaviors but outcomes did not vary by CHW dose. Although the dose of 4 CHW visits was carefully chosen based on effective doses with other studies [6], the dose and intensity may ultimately have been insufficient to influence participant psychosocial factors and other outcomes. The main trial was powered to show changes in oral health behaviors, and therefore power may not be adequate for this secondary analysis.

While we did not find that an oral health CHW intervention influenced household level psychosocial factors, we do not believe this represents a failure of CHW interventions. On the contrary, we conclude that future oral health CHW interventions should more fully address social determinants of oral health to change behavioral and clinical outcomes. This study has contributed to a more nuanced discussion around psychosocial factors, such as depression and anxiety, and living with poverty and structural racism. Future work will focus more on the effective measurement of social determinants of oral health at the individual, household and neighborhood levels, which will inform multi-level interventions to change behaviors.

## Data availability statement

The original contributions presented in the study are included in the article/Supplementary material, further inquiries can be directed to the corresponding author.

## Ethics statement

The studies involving human participants were reviewed and approved by Institutional Review Boards at the University of Illinois at Chicago (2017-1090), the University of California San Francisco (16-19920), and the Chicago Department of Public Health (16-06) approved the trial. Written informed consent to participate in this study was provided by the participants' legal guardian/next of kin. Written informed consent was obtained from the individual(s) for the publication of any potentially identifiable images or data included in this article.

## Author contributions

All authors listed have made a substantial, direct, and intellectual contribution to the work and approved it for publication.

## Funding

Research reported in this publication was supported by the National Institutes of Dental and Craniofacial Research of the National Institutes of Health under Award Number UH3DE025483, Principal Investigator: MM, and Coordinating Center Award Number U01DE025507, Principal Investigator: Stuart A. Gansky, University of California, San Francisco.

## Conflict of interest

The authors declare that the research was conducted in the absence of any commercial or financial relationships that could be construed as a potential conflict of interest.

## Publisher's note

All claims expressed in this article are solely those of the authors and do not necessarily represent those of their affiliated organizations, or those of the publisher, the editors and the reviewers. Any product that may be evaluated in this article, or claim that may be made by its manufacturer, is not guaranteed or endorsed by the publisher.

## Author disclaimer

The content is solely the responsibility of the authors and does not necessarily represent the official views of the National Institutes of Health.
