# Correction: Oral community health worker-led interventions in households with average levels of psychosocial factors

**DOI:** 10.3389/froh.2025.1664267

**Published:** 2025-11-20

**Authors:** Helen H. Lee, David Avenetti, Yuwa Edomwande, Vyshiali Sundararajan, Liyong Cui, Michael Berbaum, Rachel Nordgren, Anna Sandoval, Molly A. Martin

**Affiliations:** 1 Department of Anesthesiology, University of Illinois at Chicago, Chicago, IL, United States; 2 Institute for Health Research and Policy, University of Illinois at Chicago, Chicago, IL, United States; 3 Department of Pediatric Dentistry, University of Illinois at Chicago, Chicago, IL, United States; 4 Department of Pediatrics, University of Illinois at Chicago, Chicago, IL, United States

**Keywords:** community health worker, oral health, psychosocial stress, childhood, parenting

A Correction on Oral community health worker-led interventions in households with average levels of psychosocial factors By Lee HH, Avenetti D, Edomwande Y, Sundararajan V, Cui L, Berbaum M, Nordgren R, Sandoval A and Martin MA (2022). Front. Oral. Health. 3:962849. doi: 10.3389/froh.2022.962849

There is an error in scale calculation regarding the PROMIS Social functioning variable. Recorded scores were incorrectly inverted, resulting in the final calculated scores reflecting the opposite direction of what was intended. When rerun using the correct scoring, the analysis results including this variable did not change. Our corrections are to correct the actual values reported in Table 2 and Figure 3.

There were mistakes in Table 2 as published (inserted below)

**Table:** 

	Baseline *N* = 422	6 months *N* = 366	12 months *N* = 362
PROMIS anxiety T-score, mean (SD); median (range, IQR)	46.6 (8.1); 40.3 (40.3–77.9, 13.4)	46.7 (8.4); 40.3 (40.3–81.6, 13.4)	46.9 (8.2); 40.3 (40.3–81.6, 13.4)
PROMIS depression T-score, mean (SD); median (range, IQR)	46.2 (6.9); 1.0 (41.0–71.2, 10.8)	45.7 (6.8); 41.0 (41.0–79.4, 8.0)	45.7 (6.5); 41.0 (41.0–69.4, 10.8)
PROMIS social functioning T-score, mean (SD); median (range, IQR)	32.0 (6.9); 31.3 (25.9–58.2, 10.3)	32.1 (6.7); 31.3 (25.9–55.7, 11.0)	32.7 (6.9); 31.3 (25.9–58.2, 11.8)
PROMIS emotional T-score, mean (SD); median (range, IQR)	55.9 (8.9); 57.8 (24.7–63.5, 14.3)	56.0 (8.8); 60.7 (32.5–63.5, 14.3)	56.6 (8.3); 63.5 (24.7–63.5, 14.3)
PROMIS informational T-score, mean (SD); median (range, IQR)	57.7 (9.8); 58.7 (27.1–69.1, 17.9)	58.0 (10.0); 58.7 (23.7–69.1, 19.0)	59.1 (9.5); 60.3 (31.8–69.1, 16.7)
PROMIS instrumental T-score, mean (SD); median (range, IQR)^a^	54.8 (9.3); 55.4 (31.1–65.6, 18.4)	55.2 (9.4); 55.4 (31.1–65.6, 18.4)	55.5 (9.5); 55.4 (27.0–65.6, 18.4)
CHAOS total (avg), mean (SD); median (range, IQR)	2.3 (0.6); 2.2 (1.0–4.5, 0.8)	2.3 (0.6); 2.2 (1.0–4.2, 1.0)	2.3 (0.6); 2.3 (1.0–4.3, 1.0)

Values for the PROMIS Social Functioning T-scores, mean (SD), median (range, IQR) were incorrect, as well as the N for Baseline.

The corrected Table 2 appears below.

**Table:** 

	Baseline *N* = 420	6 months *N* = 366	12 months *N* = 362
PROMIS anxiety T-score, mean (SD); median (range, IQR)	46.6 (8.1); 40.3 (40.3–77.9, 13.4)	46.7 (8.4); 40.3 (40.3–81.6, 13.4)	46.9 (8.2); 40.3 (40.3–81.6, 13.4)
PROMIS depression T-score, mean (SD); median (range, IQR)	46.2 (6.9); 41.0 (41.0–71.2, 10.8)	45.7 (6.8); 41.0 (41.0–79.4, 8.0)	45.7 (6.5); 41.0 (41.0–69.4, 10.8)
PROMIS social functioning T-score, mean (SD); median (range, IQR)	58.0 (8.1); 58.2 (31.3–65.4, 12.7)	57.8 (8.0); 58.2 (33.6–65.4, 13.7)	57.1 (8.1); 58.2 (31.3–65.4, 14.6)
PROMIS emotional T-score, mean (SD); median (range, IQR)	55.9 (8.9); 57.8 (24.7–63.5, 14.3)	56.0 (8.8); 60.7 (32.5–63.5, 14.3)	56.6 (8.3); 63.5 (24.7–63.5, 14.3)
PROMIS informational T-score, mean (SD); median (range, IQR)	57.7 (9.8); 58.7 (27.1–69.1, 17.9)	58.0 (10.0); 58.7 (23.7–69.1, 19.0)	59.1 (9.5); 60.3 (31.8–69.1, 16.7)
PROMIS instrumental T-score, mean (SD); median (range, IQR)^a^	54.8 (9.3); 55.4 (31.1–65.6, 18.4)	55.2 (9.4); 55.4 (31.1–65.6, 18.4)	55.5 (9.5); 55.4 (27.0–65.6, 18.4)
CHAOS total (avg), mean (SD); median (range, IQR)	2.3 (0.6); 2.2 (1.0–4.5, 0.8)	2.3 (0.6); 2.2 (1.0–4.2, 1.0)	2.3 (0.6); 2.3 (1.0–4.3, 1.0)

There was a mistake in the values for Figure 3 as published.

Values for the Y-axis, “PROMIS Functioning T-score” were incorrect.

The corrected caption of Figure 3 appears below.

**Figure 3:**
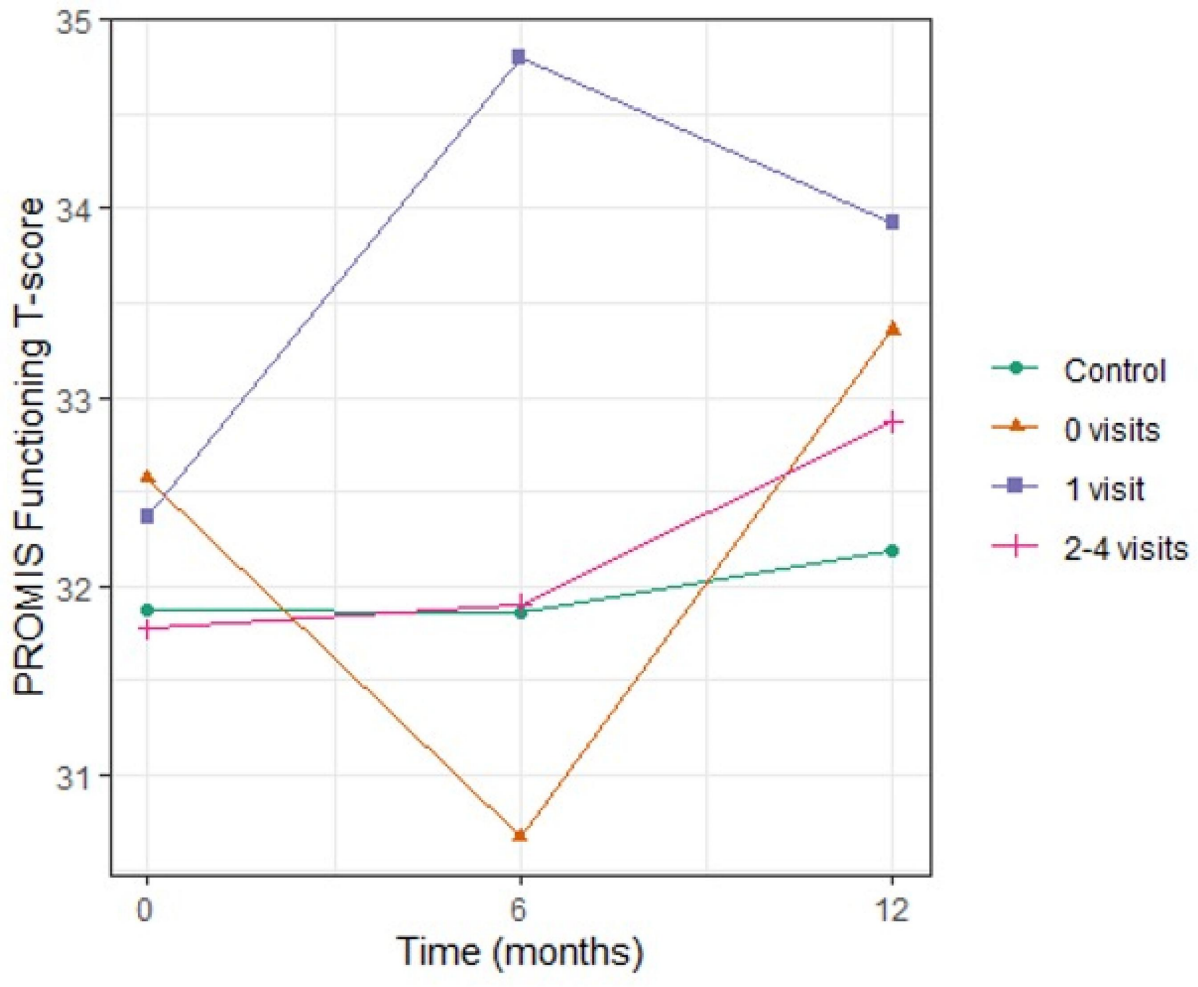


**Figure 3:**
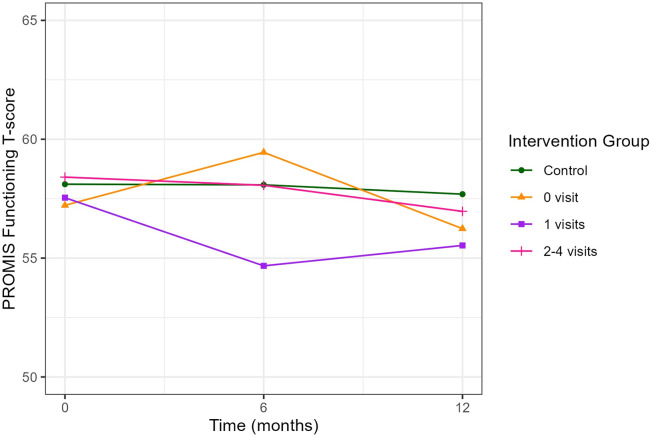


Social Functioning scores were incorrectly reported.

A correction has been made to the section: Results, Psychosocial Factors, Paragraph 1. The correct text appears below:

“Psychosocial factor levels did not vary over time (Table 2) or differ by arm. Stress and social support levels were comparable to the general population, (31) except for social functioning. CO-OP caregivers reported social functioning levels slightly above the general population average [58.0 (SD 8.1), 57.8 (8.0), and 57.1 (8.1) at 0, 6, and 12 months respectively; population mean = 50 (SD 10)]. Stratifying social functioning by CHW dose (number of visits) did not reveal a dose effect; participants who had zero (56.2, SD 8.0), one (55.5, SD 7.5), or two to four (57.0, SD 8.7) visits all had similar mean social functioning at 12 months compared to the control group (57.7, SD 8.0) (Figure 3). As there was no variation in psychosocial variables, we did not conduct further analyses”.

The original version of this article has been updated.

