# Supplementary material for: Oral community health worker-led interventions in households with average levels of psychosocial factors
Source: Front Oral Health. 2022 Aug 11;3:962849. doi: 10.3389/froh.2022.962849 (PMC9403266; doi:10.3389/froh.2022.962849)
Supplement: Supplementary file 1 [file Data_Sheet_1.docx]

Topics Covered in CHW-led Intervention Visits

**Table 1. Oral Health Basics**

| **Completed Visits** | **Total participants received, # (%)**  **N=171** | **Visit 1 participants received, # (%)**  **N=153** | **Visit 2 participants received, # (%)**  **N=84** | **Visit 3 participants received, # (%)**  **N=114** | **Visit 4 participants received, # (%)**  **N=69** |
| --- | --- | --- | --- | --- | --- |
| **Any Oral Health Basics (total)*** | **169 (98.8)** | **150 (98.0)** | **48 (57.1)** | **76 (66.7)** | **55 (79.7)** |
| **Oral Health Subtopics**** |  | | | | |
| Normal tooth development | 136 (80.5) | 118 (78.7) | 21 (43.8) | 32 (42.1) | 23 (41.8) |
| Tooth anatomy | 138 (81.7) | 112 (74.7) | 15 (31.3) | 31 (40.8) | 24 (43.6) |
| What are caries | 163 (96.4) | 140 (93.3) | 28 (58.3) | 51 (67.1) | 36 (65.5) |
| Causes of caries | 164 (97.0) | 141 (94.0) | 32 (66.7) | 60 (78.9) | 41 (74.5) |
| Importance of caries prevention | 165 (97.6) | 143 (95.3) | 40 (83.3) | 62 (81.6) | 50 (90.9) |
| Other | 5 (3.0) | 0 (0.0) | 3 (6.3) | 1 (1.3) | 1 (1.4) |

*Percent denominator is number of visits completed per category

**Percent denominator is “total” row per column

Other=plaque, basics review

**Table 2. Tooth Brushing**

| **Completed Visits** | **Total participants received, # (%)**  **N=171** | **Visit 1 participants received, # (%)**  **N=153** | **Visit 2 participants received, # (%)**  **N=84** | **Visit 3 participants received, # (%)**  **N=114** | **Visit 4 participants received, # (%)**  **N=69** |
| --- | --- | --- | --- | --- | --- |
| **Any Tooth Brushing (total)*** | **169 (98.8)** | **151 (98.7)** | **63 (75.0)** | **104 (91.2)** | **64 (92.8)** |
| **Tooth Brushing Subtopics**** |  | | | | |
| When to start | 107 (63.3) | 77 (51.0) | 15 (23.8) | 43 (41.3) | 10 (15.6) |
| Frequency | 159 (94.1) | 134 (88.7) | 54 (85.7) | 98 (94.2) | 53 (82.8) |
| Technique | 153 (90.5) | 127 (84.1) | 38 (60.3) | 84 (80.8) | 46 (71.9) |
| Duration | 157 (92.9) | 133 (88.1) | 48 (76.2) | 87 (83.7) | 50 (78.1) |
| Equipment | 127 (75.1) | 96 (63.6) | 33 (52.4) | 66 (63.5) | 41 (64.1) |
| Toothpaste amount | 154 (91.1) | 119 (78.8) | 41 (65.1) | 68 (65.4) | 43 (67.2) |
| Spitting | 135 (79.9) | 85 (56.3) | 32 (50.8) | 64 (61.5) | 41 (64.1) |
| Flossing | 158 (93.5) | 132 (87.4) | 39 (61.9) | 73 (70.2) | 45 (70.3) |
| Other*** | 15 (8.9) | 2 (1.3) | 7 (11.1) | 0 (0.0) | 6 (9.4) |

*Percent denominator is number of visits completed per category

**Percent denominator is “total” row per column

*** Antibacterial mouthwash, locating TB resources, participant and household-specific brushing, wiping, TB review, equipment storage

**Table 3. Fluoride**

| **Completed Visits** | **Total participants received, # (%)**  **N=171** | **Visit 1 participants received, # (%)**  **N=153** | **Visit 2 participants received, # (%)**  **N=84** | **Visit 3 participants received, # (%)**  **N=114** | **Visit 4 participants received, # (%)**  **N=69** |
| --- | --- | --- | --- | --- | --- |
| **Any Fluoride (total)*** | **169 (98.8)** | **152 (99.3)** | **51 (60.7)** | **76 (66.7)** | **45 (65.2)** |
| **Fluoride Subtopics**** |  | | | | |
| Define fluoride and roles | 145 (85.8) | 125 (82.2) | 26 (51.0) | 47 (61.8) | 20 (44.4) |
| Toothpaste | 168 (99.4) | 150 (98.7) | 43 (84.3) | 72 (94.7) | 42 (93.3) |
| Water | 169 (100.0) | 150 (98.7) | 40 (78.4) | 73 (96.1) | 39 (86.7) |
| Varnish | 165 (97.6) | 141 (92.8) | 35 (68.6) | 69 (90.8) | 32 (71.1) |
| Fluoride risks/myths | 80 (47.3) | 37 (24.3) | 14 (27.5) | 34 (44.7) | 5 (11.1) |
| Other*** | 14 (8.3) | 9 (5.9) | 2 (3.9) | 1 (1.3) | 3 (6.7) |

*Percent denominator is number of visits completed per category

**Percent denominator is “total” row per column

*** Pediatric dentist as a fluoride provider, fluoridated mouth rinse, fluoride sources

**Table 4. Weaning**

| **Completed Visits** | **Total participants received, # (%)**  **N=171** | **Visit 1 participants received, # (%)**  **N=153** | **Visit 2 participants received, # (%)**  **N=84** | **Visit 3 participants received, # (%)**  **N=114** | **Visit 4 participants received, # (%)**  **N=69** |
| --- | --- | --- | --- | --- | --- |
| **Any Weaning (total)*** | **149 (87.1)** | **116 (75.8)** | **41 (48.8)** | **72 (63.2)** | **30 (43.5)** |
| **Weaning Subtopics**** |  | | | | |
| When to transition from bottle | 98 (65.8) | 79 (68.1) | 15 (36.6) | 32 (44.4) | 9 (30.0) |
| Sippy cup recommendations | 133 (89.3) | 97 (83.6) | 26 (63.4) | 60 (83.3) | 25 (83.3) |
| Nighttime feeding | 141 (94.6) | 102 (87.9) | 38 (92.7) | 66 (91.7) | 26 (86.7) |
| Other*** | 10 (6.7) | 3 (2.6) | 4 (9.8) | 2 (2.8) | 1 (3.3) |

*Percent denominator is number of visits completed per category

**Percent denominator is “total” row per column

*** Baby formula, diluting method, pacifier, defining weaning, participant-specific weaning, breastfeeding, naptime feeding

**Table 5. Nutrition**

| **Completed Visits** | **Total participants received, # (%)**  **N=171** | **Visit 1 participants received, # (%)**  **N=153** | **Visit 2 participants received, # (%)**  **N=84** | **Visit 3 participants received, # (%)**  **N=114** | **Visit 4 participants received, # (%)**  **N=69** |
| --- | --- | --- | --- | --- | --- |
| **Any Nutrition (total)*** | **152 (88.9)** | **101 (66.0)** | **77 (91.7)** | **85 (74.6)** | **55 (79.7)** |
| **Nutrition Subtopics**** |  | | | | |
| Sugar-sweetened beverages | 139 (91.4) | 91 (90.1) | 54 (70.1) | 64 (75.3) | 38 (69.1) |
| Juice | 141 (92.7) | 91 (90.1) | 59 (76.6) | 60 (70.6) | 38 (69.1) |
| Sticky foods, candy, etc | 140 (92.1) | 84 (83.2) | 62 (80.5) | 63 (74.1) | 42 (76.4) |
| Frequency of foods/drinks | 148 (97.4) | 90 (89.1) | 61 (79.2) | 64 (75.3) | 45 (81.8) |
| Healthy foods | 139 (91.4) | 71 (70.3) | 72 (93.5) | 72 (84.7) | 54 (98.2) |
| Other*** | 7 (4.6) | 3 (3.0) | 2 (2.6) | 2 (2.4) | 0 (0.0) |

*Percent denominator is number of visits completed per category

**Percent denominator is “total” row per column

*** Understanding snack labels, baby formula, participant-specific nutrition information, soda, fast food

**Table 6. Seeing the Dentist**

| **Completed Visits** | **Total participants received, # (%)**  **N=171** | **Visit 1 participants received, # (%)**  **N=153** | **Visit 2 participants received, # (%)**  **N=84** | **Visit 3 participants received, # (%)**  **N=114** | **Visit 4 participants received, # (%)**  **N=69** |
| --- | --- | --- | --- | --- | --- |
| **Any Seeing the Dentist (total)*** | **163 (95.3)** | **140 (91.5)** | **62 (73.8)** | **98 (86.0)** | **52 (75.4)** |
| **Seeing the Dentist Subtopics**** |  |  |  |  |  |
| When to start | 123 (75.5) | 100 (71.4) | 13 (21.0) | 39 (39.8) | 10 (19.2) |
| What to expect | 125 (76.7) | 83 (59.3) | 23 (37.1) | 57 (58.2) | 20 (38.5) |
| Frequency | 156 (95.7) | 127 (90.7) | 39 (62.9) | 87 (88.8) | 42 (80.8) |
| Role of the pediatrician | 96 (58.9) | 55 (39.3) | 10 (16.1) | 38 (38.8) | 12 (23.1) |
| Other*** | 57 (35.0) | 12 (8.6) | 27 (43.5) | 17 (17.3) | 16 (30.8) |

*Percent denominator is number of visits completed per category

**Percent denominator is “total” row per column

***Fluoride supplement, assistance with and progress of dental appointments/referrals, insurance coverage, dental resources for those uninsured, barriers to dentist visits, CG dental care, participant’s dental experiences, cavities

**Table 7. Other Topics**

| **Completed Visits** | **Total participants received, # (%)**  **N=171** | **Visit 1 participants received, # (%)**  **N=153** | **Visit 2 participants received, # (%)**  **N=84** | **Visit 3 participants received, # (%)**  **N=114** | **Visit 4 participants received, # (%)**  **N=69** |
| --- | --- | --- | --- | --- | --- |
| **Any Other Topics (total)*** | **132 (77.2)** | **74 (48.4)** | **55 (65.5)** | **84 (73.7)** | **48 (69.6)** |
| **Subtopics**** | **130 (98.4)** | **74 (100.0)** | **55 (100.0)** | **80 (95.2)** | **48 (100.0)** |

*Percent denominator is number of visits completed per category

**Percent denominator is “total” row per column. Open text field with 130 responses: Insurance coverage, immigration, financial assistance, Link benefits, English as second language, caregiver dental concerns, child education, mental health, continuing education for caregivers, housing, childcare, transportation, employment, nutrition, health/medical concerns, child support, physical activity, social resources
